# The impact of survey mode on the response rate in a survey of the factors that influence Minnesota physicians’ disclosure practices

**DOI:** 10.1186/s12874-019-0719-7

**Published:** 2019-04-02

**Authors:** Lesley Weaver, Timothy J. Beebe, Todd Rockwood

**Affiliations:** Division of Health Policy and Management, University of Minnesota, 420 Delaware Street Southeast, MMC 729, Minneapolis, MN 55455 USA

**Keywords:** Mixed-mode, Physicians, Response rate, Non-response bias

## Abstract

**Background:**

There is evidence that the physician response rate is declining. In response to this, methods for increasing the physician response rate are currently being explored. This paper examines the response rate and extent of non-response bias in a mixed-mode study of Minnesota physicians.

**Methods:**

This mode experiment was embedded in a survey study on the factors that influence physicians’ willingness to disclose medical errors and adverse events to patients and their families. Physicians were randomly selected from a list of licensed physicians obtained from the Minnesota Board of Medical Practice. Afterwards, they were randomly assigned to either a single-mode (mail-only or web-only) or mixed-mode (web-mail or mail-web) design. Differences in response rate and nonresponse bias were assessed using Fischer’s Exact Test.

**Results:**

The overall response rate was 18.60%. There were no statistically significant differences in the response rate across modes (p – value = 0.410). The non-response analysis indicates that responders and non-responders did not differ with respect to speciality or practice location.

**Conclusions:**

The mode of administration did not affect the physician response rate.

## Background

Surveys are a useful means of collecting information on physicians’ knowledge, attitudes, and beliefs. Unfortunately, the physician response rate is declining [1–4], threatening the external validity of physician surveys and increasing the possibility of non-response bias. However, prior research suggests that physician surveys may be less prone to non-response bias than surveys of other populations, given that physicians are rather homogenous with respect to their knowledge, attitudes, and beliefs [5]. Nevertheless, researchers are searching for effective ways of increasing their participation in surveys, given that the response rate is often considered an indicator of survey quality. Specifically, the greater a survey’s response rate; the greater the study’s external validity [6].

Prior research suggests numerous strategies that could be used to increase the physician response rate, including the use of incentives [7], short questionnaires [5, 8], multiple reminders [9, 10], and survey sponsorship [11, 12]. Monetary incentives tend to be more effective at increasing the response rate than non-monetary incentives and lotteries [11]. And, prepaid incentives tend to work better than promised incentives [7, 11]. However, a study conducted by Ziegenfuss, Niederhauser, Kallmes, and Beebe [13] found that responders preferred the chance to win an iPad to the guarantee of receiving a $5 Amazon giftcard. Sending multiple reminders is particularly important, given physicians’ busy schedules and demanding workloads, which can lead to refusals and unit non-response.

The mode, or medium used to administer the questions to potential respondents, can also affect the response rate [14]. Compared to web surveys, physicians are more apt to respond to mail surveys [7, 11, 15]. And, the use of mixed-mode designs tends to generate a higher response rate amongst health care professionals than single-mode designs [16–19]. However, single-mode designs tend to generate a higher response rate than simultaneous, mixed-mode designs [20]. Mixed-mode designs allow physicians to choose the mode they will use to respond to a survey request. The availability of mode choice may be particularly important to physicians, given that they are accustomed to having considerable autonomy in their professional lives.

With mixed-mode designs, the sequencing and timing of the medium used to administer the survey is important. Beebe and colleagues [21] found that following a mail survey with a web survey produces a higher response rate amongst physicians than doing the opposite. And, a meta-analysis conducted by Medway and Fulton [20] found that sequential, mixed-mode designs tend to produce a higher response rate than simultaneous, mixed-mode designs. However, their analysis was based on studies of various populations, so their results may not be generalizable to physicians. For simultaneous, mixed-mode designs, the rate maybe lower because asking individuals to make a choice places an additional response burden on them. For instance, in a web-mail design, they might spend time weighing the advantages and disadvantages of each option. And, if they choose the web option, they must find an Internet connected device, open a web browser, and type in the survey link.

In some mixed-mode studies of physicians, the final mode used differs from the mode used for all the prior contacts [21–25]. However, one study did change modes after the initial mailing [19]. This mixed-mode study combines elements of the aforementioned designs to examine which mode of contact has the greatest impact on the physician response rate. It also looks at their impacts on nonresponse bias (i.e., the extent to which responders differ from non-responders).

### Current study

Prior research suggests that physicians are more apt to respond to mail surveys than web surveys [7, 11, 15]. However, the practice of medicine is becoming more technologically driven. For instance, many hospitals and clinics have transitioned from paper medical records to electronic medical records, requiring physicians to use computers as part of their day-to-day practice. Based on this, one could assume that they are comfortable using them. If this is indeed the case, then perhaps the current generation of physicians will be more receptive to completing a web survey than their predecessors. The purpose of this cross-sectional, mixed-mode study is to examine how the mode of survey administration affects the physician response rate.

## Methods

The mode experiment was embedded in The Medical Error Disclosure Survey (MEDS) and the Adverse Event Disclosure Survey (AEDS), which was fielded from November 2017 to February 2018. They examined the factors that influence physicians’ willingness to disclose medical errors and adverse events to patients and their families, respectively.

A list of 14,772 licensed, Minnesota physicians was obtained from the Minnesota Board of Medical Practice. From this list, a random sample of 1565 physicians was selected. Of those selected, 341 (21.79%) only had a postal address listed. The remaining 1224 (78.21%) had both a postal and email address listed. Physicians in the latter group were randomly assigned to one of four mode groups: mail-only, mail-web, web-mail, and web-only. There were 306 physicians in each group. Within each mode group, physicians were randomly assigned to receive either the MEDS (*n* = 612) or AEDS (*n* = 612). Of these, 293 physicians participated in the survey, yielding an unweighted response rate of 18.60%.

**Fig. 1:**
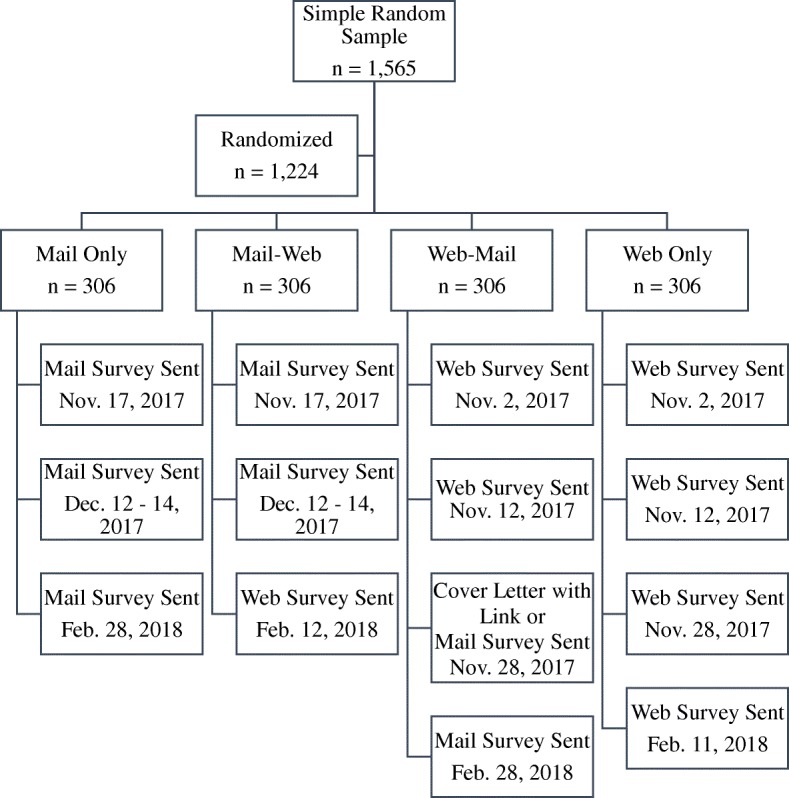
Data collection and mode assignment

Figure 1 depicts the crossover design used in this study. All the mail contacts included a cover letter that was printed on the University of Minnesota, Twin Cities letterhead. The letter explained the purpose of the study, why they were selected, and the voluntary, confidential nature of their participation. It was accompanied by a copy of their assigned survey booklet and a business reply envelope. The paper surveys were returned to the primary author at the School of Public Health at the University of Minnesota, Twin Cities. At the end of data collection, the surveys were given to Northwest Keypunch, Inc., where they were entered into a database by data entry professionals. Upon return of the surveys and receipt of the database, the primary author randomly spot-checked the data to ensure its accuracy.

For all web surveys, the body of the email included information that was similar to what was included in the mailed cover letters. The emails also included an embedded link to the survey, which were programmed using Qualtrics™. At the end of data collection, an Excel file containing participants’ responses was downloaded from Qualtrics. It was merged with the database from Northwest Keypunch, Inc. prior to data analysis.

Initially, physicians in the web-mail group were informed of the survey via email. At first, non-responders were sent an email reminder, which included a link to the survey. Physicians who did not respond to that email were randomly assigned to one of two groups—a reminder letter or survey packet group. Those in the reminder group were mailed a reminder letter containing a personalized, survey link, which they were asked to type into their internet browser. Meanwhile, those in the survey packet group were mailed a cover letter, survey booklet, and business reply envelope. Later, non-responders in both groups were sent a survey packet.

Non-responders in the mail-only and mail-web groups received up to two additional contacts. In contrast, non-responders in the web-mail and web-only groups received up to three additional contacts. When physicians returned the survey, refused to participate, or were deemed ineligible, all subsequent contact with them ceased. Informed consent was implied if physicians completed and returned the survey. Written and verbal consent was not obtained. Physicians who completed the survey were entered into a drawing for their choice of one of four tablets (market value approximately $500). This study was approved by the Institutional Review Board at the University of Minnesota, Twin Cities.

### Analysis

By mode, response rates were computed by tallying the number of completes and dividing it by the number of eligible cases in accordance with the RR1 guidelines outlined by the American Association for Public Opinion Research [26]. The chi-square test was used to test for overall differences in the response rate across modes, and the Fisher’s Exact test was used to examine potential non-response bias. Data from the original sampling frame was used to compare the practice area and location of responders and non-responders within each group. To determine location, the sampling frame was merged with the 2004 ZIP RUCA Code files for the state of Minnesota, which was obtained from the Washington, Wyoming, Alaska, Montana and Idaho (WWAMI) Rural Health Research Center [27]. Unfortunately, it was not possible to compare responders and non-responders on other variables because the sampling frame only included physicians’ license number, specialty, and mailing address and/or email address. A *p*-value of 0.05 was used to determine statistical significance. All analyses were conducted using STATA, Version 15 [28].

## Results

For the mode experiment, the overall response rate was 18.60%. Table 1 presents the response rates by mode. The mail-only and mail-web groups had the highest response rate at 19%. The web-only group had the lowest at 15%. However, these results were not statistically significant.

**Table 1:** Response rate by mode

	Respondents	Non-respondents	X^2^	P-value
Mail Only	18.96% (62)	81.04% (265)		
Mail-Web	18.95% (58)	81.05% (248)		
Web-Mail (link)	20.12% (34)	79.88% (135)	3.9726	0.410
Web-Mail (booklet)	22.15% (35)	77.85% (123)		
Web Only	15.21% (47)	84.79% (262)		
Total	18.60% (236)	81.40% (1033)		

Table 2 compares the practice area of responders and non-responders by mode. Across all modes, the majority of responders were specialists. The proportion of responders who were specialists ranged from 45.71% in the web-mail (booklet) group to 73.53% in the web-mail (link) group. Across all modes, there were not any statistically significant differences in the practice area of responders and non-responders.

**Table 2:** Practice area of responders and non-responders

	Responders		Non-responders	
	Generalist Practice	Specialist Practice	Generalist Practice	Specialist Practice
Mail Only	45.16% (28)	54.84% (34)	48.68% (129)	51.32% (136)
Mail-Web	43.10% (25)	56.90% (33)	45.16% (112)	54.84% (136)
Web-Mail (link)	26.47% (9)	73.53% (25)	45.93% (62)	54.07% (73)
Web-Mail (booklet)	54.29% (19)	45.71% (16)	45.53% (56)	54.47% (67)
Web Only	42.55% (20)	57.45% (27)	50.76% (133)	49.24% (129)
Total	42.80% (101)	57.20% (135)	47.63% (492)	52.37% (541)
P-value	0.217		0.714	

Notes: Generalist practice include physicians practicing in one or more of the following areas: Emergency Medicine, Family Medicine, and Internal Medicine. Specialist practice includes physicians practicing in one or more of the following areas: Allergy and Immunology, Anesthesiology, Colon and Rectal Surgery, Dermatology, Medical Genetics and Genomics, Neurological Surgery, Nuclear Medicine, Obstetrics and Gynecology, Ophthalmology, Otolaryngology, Orthopedic Surgery, Pathology, Pediatrics, Physical Medicine and Rehabilitation, Plastic Surgery, Preventative Medicine, Psychiatry and Neurology, Radiology, Surgery, Thoracic Surgery, and Urology

Table 3 compares the practice location of responders and non-responders by mode. Regardless of mode, the majority of them practice in an urban area. There were not any statistically significant differences in practice location amongst the two groups.

**Table 3:** Practice location of responders and non-responders

	Responders			Non-responders		
	Urban	Large Rural	Small Rural	Urban	Large Rural	Small Rural
Mail Only	81.97% (50)	13.11% (8)	4.92% (3)	85.61% (226)	9.47% (25)	4.92% (13)
Mail-Web	89.66% (52)	8.62% (5)	1.72% (1)	88.71% (220)	7.66% (19)	3.63% (9)
Web-Mail (link)	82.35% (28)	11.76% (6)	5.88% (2)	85.93% (116)	8.89% (12)	5.19% (7)
Web-Mail (booklet)	71.43% (25)	17.14% (6)	11.43% (4)	83.74% (103)	9.76% (12)	6.50% (8)
Web Only	93.62% (44)	4.26% (2)	2.13% (1)	87.40% (229)	9.92% (26)	2.67% (7)
Total	84.68% (199)	10.64% (27)	4.68% (9)	86.63% (894)	9.11% (94)	4.26% (44)
P-value	0.223			0.735		

## Discussion

There were not any statistically significant differences in the response rate across modes. The higher response rate for the web-mail group was unexpected, but consistent with prior research [19]. However, the finding that the overall response rate was lowest for the web-only group was expected. Amongst physicians the response rate for mailed surveys tend to be greater than it is for web surveys [3, 7, 11, 15, 17, 29]. There could be numerous reasons for this. Due to spam filters, the emailed invitations could have ended up in physicians’ spam folders only to be deleted later. Also, the volume of emails that some physicians receive may force them to skim their inboxes and only respond to the most important emails. It was not possible to determine if physicians deleted the email invitations without opening them or if they were diverted by spam filters.

Given specialists’ demanding work schedules, it was surprising to find that they had a higher response rate than generalists, especially for the web-only group. In a study comparing mail and web surveys, Leece and colleagues [15] found that surgeons who are members of the Orthopaedic Trauma Association are more apt to respond to mail surveys than web surveys. And, in a study of various specialists, Cunningham and colleagues [30] found that the response rate to their web survey varied by specialty. The response rate was 46.6% for neurologists/neurosurgeons, 29.2% for pediatricians, and 29.6% for general surgeons. Taken together, these findings suggest that perhaps researchers should be using different modes when studying different groups of specialists. Future research should tease out the relationship between physicians’ specialty and their mode preferences.

Prior research suggests that individuals are more apt to respond to survey on topics that are important or of interest to them [31, 32]. Thus, it is possible that the higher response rate amongst specialists is due to the topic’s salience, not their mode preferences. Compared to generalists, specialists are apt to treat patients with multiple health conditions or that require intensive, complex medical care. Due to the complexities of care, the best laid plans for the optimal delivery of care may not pan out, leading to a medical error or series of errors. Nationwide, there is a push for the timely disclosure of medical errors to patients and/or their families, especially in hospitals. The saliency of disclosure for specialists may have prompted some of them to complete the survey. While patients treated by generalists can also experience a medical error, the issue may be less salient for them.

Additionally, the disclosure of adverse events and medical errors is a sensitive topic for many physicians. Following a medical error, physicians may experience emotional and psychological distress in the form of anxiety, sleeplessness, guilt, feelings of inadequacy, and decreased job satisfaction [33–36]. To avoid triggering unpleasant emotional or psychological states, physicians may have opted out of or refused to complete the survey. To reduce the chances of this occurring, the survey did not ask physicians to describe any medical errors or adverse events they were personally involved in. Instead, it was designed to capture their general attitudes toward the disclosure of them. However, the design of the survey and use of lottery incentives may not have been enough to overcome some physicians’ reluctance to discuss such a sensitive topic.

There are a few limitations associated with this study. First, the state licensure database did not contain many demographic variables, so it was not possible to conduct a more thorough non-response analysis. Second, the sample size was relatively small, making it difficult to identity significant differences. Third, the overall response rate was lower than the community standard of 50% for physician surveys [37], likely due to the time of year the study was conducted. The bulk of the surveys were administered around the Thanksgiving and Christmas holidays, a very busy time of year. Nevertheless, the primary purpose of the investigation was to assess the differential impact of our manipulations across conditions. While lower than expected participation may have adversely impacted the study’s statistical power and contributed to the lack of statistically significant differences, the impacts observed do offer incremental evidence in support of certain approaches to increasing survey yield. Lastly, the mode groups did not receive the same number of follow-ups, which could have influenced the response rate. Due to resource constraints, it was not possible to conduct additional follow-ups with the mail and mail-web groups.

## Conclusion

While the results presented were not statistically significant, additional research on the impact of survey mode on the physician response rate is needed. Identifying ways to improve their response rate is important, given that a low response rate can contribute to non-response bias, an unrepresentative sample, and negatively impact the generalizability of a study’s findings [6]. Future research should examine whether there is a relationship between survey mode, physicians’ specialty, and the response rate. Examining this relationship could help researchers develop more effective survey protocols in the future.

## Abbreviations

AEDS: Adverse Event Disclosure Survey
MEDS: Medical Error Disclosure Survey
WWAMI: Washington, Wyoming, Alaska, Montana and Idaho Rural Health Research Center
